# Turning to Service Users for the Understanding of Current and Future Mental Health Services in the Development Process of Research and Practice: A Qualitative Study

**DOI:** 10.1111/hex.70574

**Published:** 2026-01-24

**Authors:** Emmy Nilsson, Carina Tjörnstrand, Daniel Lindqvist, Jenny Wetterling, Annika Lexén, Ulrika Bejerholm

**Affiliations:** ^1^ Department of Health Sciences, Mental Health, Activity and Participation Lund University Lund Sweden; ^2^ Centre of Evidence‐based Psychosocial Interventions, CEPI Lund University Lund Sweden; ^3^ Department of Psychiatry Skåne University Hospital Lund Sweden; ^4^ Department of Clinical Sciences Lund, Unit for Biological and Precision Psychiatry Lund University Lund Sweden; ^5^ The ReLife Centre (The Centre for Mental Health and Recovery Across the Life Course for Persons with Serious Mental Illnesses) Lund University Lund Sweden

**Keywords:** community mental health services, evidence‐based practice, experience life, Flexible Assertive Community Treatment, mental health services, psychiatry

## Abstract

**Background:**

A person‐centred and recovery‐oriented approach is an integral part of modern mental health services founded on the experiential knowledge of service users. Their reflections as experts, grounded in their unique experience as service users, play a significant role in developing and improving the existing care. Experiential knowledge is therefore a means to enhance the relevance of research, inform the development of care, and bridge research and practice.

**Aim:**

To understand service users' experiences of their current mental health services and explore reflections on the Flexible Assertive Community Treatment (FACT) model and its role in future practice.

**Methods:**

A total of 17 experts participated in individual and dyadic in‐depth interviews. A reflexive thematic analysis was performed on the participants' experiences of current mental health services and on their reflections on a vignette describing an integrative, recovery‐oriented care and support model—FACT.

**Results:**

The analysis resulted in three themes. The first theme, ‘Losing value and credibility as a person when becoming a service user’, reflected participants' experiences of being reduced to the signs and symptoms of their mental health problems. The second theme, ‘Navigating through the mental health maze’, describes participants view on the current mental health services, while the last theme, ‘Involving service users in their care and support would be empowering’, holds participants' views on the importance of greater involvement in future service design and delivery.

**Conclusions:**

These results underscore the necessity for enhanced collaboration to empower and provide inclusive, tailored care and support, which the participants emphasised as essential for the future of mental health services. The participants reflected on certain structural concepts, such as hierarchy, caring culture, and financial strains, prior to the implementation of FACT, which need to be addressed before an adaptation of integrative, recovery‐oriented care and support models.

**Public and Service User Contribution:**

The study planning and process involved stakeholders, including user organisations, Swedish Partnership for Mental Health (NSPH), Skåne and their sister organisation LIBRA Skåne, as well as managers and professionals of mental health services. One of the authors has own experience of mental illness and contributed greatly to the data analysis and the finalising of the manuscript, and two authors have experience as relatives.

**Abbreviations**
FACT: Flexible Assertive Community Treatment
LU: Lund University
MHP: mental health problems
MHS: mental health services
RTARG: Reflexive Thematic Analysis Reporting Guidelines

## 1 Introduction

There is a risk that experiential knowledge, i.e., experience‐based knowledge, is viewed as inferior to the traditional scientific objective truth, dominated by researchers within the medical field [1]. Experiential knowledge of service users has been described differently depending on the context and culture, such as user involvement, patient engagement, patient participation etc, and its contribution to the medical field is considered to vary [2, 3, 4]. Service user engagement in research is defined as a collaborative interaction and decision‐making between service users and researchers [5]. It is seen as an active and meaningful process across all stages of the research process and users are recognised for their unique value and expertise based on their specific experience. However, service users' experiential knowledge has received little recognition from first line managers and health professionals with traditional attitudes and views of service users [6]. It is, however, essential to understand the experience of service users within the field of person‐centred and recovery‐oriented mental health services (MHS) when aiming to develop knowledge to improve the existing care and support.

It is postulated in the ‘Good Quality—Local Health Care’ reform in Sweden [7] that service users receive good, accessible and integrated care that supports health, which is the emphasis in person‐centred care. Such care is grounded on its ethics, viewing a person as a capable human being [8, 9, 10]. Policies in many countries emphasise recovery‐oriented MHS [11, 12], which is also referred to as person‐centred and growth‐oriented care [13]. Recovery in this sense does not mean returning to a previous state for the service user but rather embarking on a personal recovery journey [14]. The personal recovery journey is a process, involving; Connectedness; Hope and optimism about the future; Identity; Meaning in life; and Empowerment. This contrasts with clinical recovery, which focuses on reducing the signs and symptoms of mental illness [15]. A structural and organisational capacity of how to support mental health, as opposed to only dealing with service users' mental disease, is therefore needed. Flexible Assertive Community Treatment (FACT) helps to organise and promote continuity and flexibility in care and support that facilitates the users' recovery journey [16, 17, 18]. However, there is a need to turn to service users' experiential knowledge to understand and review the current organisational capacity to meet service user needs [19, 20] and to inform the future research and practice of FACT.

Previous research on users' experiential knowledge of seeking care and support reveals that encountering MHS can be challenging [21, 22, 23, 24]. The users' expectations included a desire for both personal and clinical recovery, with the hope of regaining their health when seeking care and support [25]. However, they frequently expressed a fear of encountering various forms of mental health stigma [20, 22, 26, 27]. The quality of encounters in MHS depended largely on the mental health literacy of the health professionals, leading to varying outcomes for the service users [24, 28]. The relationship between service users and professionals is central to the experience of quality care and support [16, 22, 23, 24, 29]. Its character varies by context and culture, and may be described as *genuine, interpersonal, therapeutic*, or *caring*, often framed as a *partnership* or *alliance* [30, 31, 32]. Service users felt that professionals did not always empower them to make decisions about their care [33] and jumped to conclusions before the service user had described their concerns. They also experienced that health professionals overmedicalised their symptoms and signs of mental health problems (MHP) [34]. To inform the development of MHS through the evidence‐based practice of health professionals, it is important to include the service users' preferences based on the literature on experiential knowledge, their clinical state and circumstances together with research evidence [35]. Government policies on mental health and suicide prevention in Sweden and elsewhere emphasise evidence‐based interventions [36], but experiential knowledge with regards to how MHS are perceived by service users and how they view and express their needs for future interventions and organisation of care is lacking.

The accessibility and acceptance of MHS by service users are recognised as indicators of quality [37]. Continuity is emphasised as crucial when asking service users with complex needs of care and support [27, 29]. In fact, continuity of care is perceived to be fundamental for sustainable mental health among service users [29, 38]. However, accessing and maintaining continuity of MHS across different providers has been perceived as challenging, for both service users and professionals [38, 39, 40]. Furthermore, quality care and support have been identified as involving individually coordinated care that is measurable, as well as the role and competence of health professionals [27, 41]. Implementing recovery‐oriented care and support models has the potential to reduce service users perceived stigma [42] while also improving professionals' job satisfaction [43]. There is thus an urgent need to equip healthcare systems to meet the rising need of preventive and promotive mental health interventions [20], such as integrated care, recovery‐oriented and support models.

Healthcare systems vary internationally in having different structures to meet the needs of service users. The Swedish welfare system relies on tax‐based funding and public provision, and the executive power is decentralised into 290 municipalities and 21 regional healthcare services [44]. The municipalities are responsible for the social support services and the regional healthcare services are responsible for the primary and specialised MHS. These services have been the subject of public tendering to improve accessibility and continuity according to service users' preferences [44, 45]. However, implementation research clearly shows that care and support services are already fragmented [27, 46, 47]. Diverse economic foundations and the organisational structures of services contribute to this, as well as a narrow focus on patient‐centred care, on diagnoses and medication or functional limitations [48]. Given the complexity of providing MHS and the overall goal of offering good health on equal terms [45], as well as the ‘Good Quality—Local Health Care’ reform [7], we emphasise the critical importance of incorporating service users' experiential knowledge to inform the development of new practices and research within specialist general MHS.

## 2 Aim

To explore service users' experiences of their current MHS and their reflections on the FACT model and its role in future practice.

## 3 Methods

### 3.1 Study Design

To address the research aim, a reflexive thematic analysis [49, 50] was conducted using individual and dyadic interviews [51]. Dyadic interviews are a form of interactive interviewing that offer both social interaction and depth of two participants, when conducted by a trained interviewer. The focus of the interviews was the descriptions of the experts' experiential knowledge of being a service user in MHS, and their reflections on a vignette Table 1, portraying the integrative, recovery‐oriented care and support model‐ FACT. The study had a participatory design, and the authors adhered to a critical realist position from the viewpoint of knowledge emerging from a social constructionism in the interpretation of the data [52]. This research was also conceptualised within caring at a later stage in the process. The Swedish Ethical Review Authority (Dnr 2019‐02866) approved the study, and all the participants provided informed consent at the time of the interviews.

**Table 1:** The vignette of Flexible Assertive Community Treatment (FACT) service user.

Vignette FACT‐Brukare (Swedish)	Vignette FACT user
‘*De senaste åtta åren har jag fått stöd från ett annat håll. Nu är det flexibla ACT teamet som tittar till mig. De kommer hem till mig för att prata och se hur jag har det. Och de hjälper mig att tackla saker och ting på riktigt, så att det verkligen blir av. Det betyder jättemycket att ha ett helt team runt omkring sig när man mår dåligt, människor som kan hjälpa till med alla typer av problem. Det är nästa som att vara på sjukhus fast hemma. Men fördelen är att man är i sin egen lägenhet och känner all personal. Jag lyckades komma ur svackan ganska snabbt denna gång och nu träffar jag bara min kontaktperson och återhämtningsgruppen. Allt som allt är jag ganska nöjd med min situation för tillfället’*.	‘I've received support from other people the last eight years. It's the flexible ACT team who checks on me. They come home to me to talk and see if I'm alright. And they help me to deal with things so that they get done. It means a lot to have a whole team when you feel bad, people who can help with all types of problems. It's almost like being in the hospital although I'm at home. But the benefit is that you're in your own flat and know all the staff. I managed to get out of my depression quite quickly this time and I now only meet my key worker and the recovery group. All in all, I'm quite pleased about my situation just now’.

### 3.2 Context and Setting

The present study examines the initial phase of implementing FACT within specialist general MHS in southern Sweden. The long‐term aim was to co‐produce the planning and execution of a randomised controlled trial. The initiative began in 2017 through collaboration between the regional health authority, municipalities, FACT implementation strategists, project leaders, professionals, service user organisations, and researchers (AL & UB). During the research application planning, it was agreed to include an evaluation of the implementation process, exploring experiential knowledge from service users, professionals, and managers across 3 sectors and 11 municipalities in southern Sweden. All stakeholders were invited as experts. The research proposal, *Ups & Downs in Mental Health*, was funded by FORTE (RN 2019‐01730), and this study represents the first outlined component.

### 3.3 FACT Intervention

FACT has its origin in the Netherlands [18] and has been implemented into Swedish specialist MHS [17, 53]. There is limited knowledge of the FACT model's effectiveness in a general psychiatry outpatient context and there is a need for a randomised controlled trial to evaluate its effectiveness [54]. A quasi‐experimental evaluation showed that FACT ensured the safe delivery of more intensive care and support than traditional specialist MHS [55].

### 3.4 Participants

Participants were recruited from outpatient units in general MHS. Criterion criteria required experience as a service user in these services [56]. Purposive sampling invited all potential participants as experts on general outpatient care, excluding those with experience in primary care, addiction, forensic, or psychosis services. Recruitment was facilitated through posters titled *Psychiatry for the Future* displayed in waiting rooms within sectors proposed for the anonymised trial, providing interview details. The Swedish Partnership for Mental Health Skåne, NSPH and its affiliated organisations LIBRA Balans Skåne supported recruitment by informing their members. All interested individuals were included. No personal data were collected during recruitment, as experiential knowledge was not linked to gender, age, ethnicity, or diagnosis. Initially, participants were invited to focus groups, but the process was adapted to individual or dyadic interviews to respect autonomy. Contact details were used only for scheduling and then deleted. At the start of interviews, participants received standardised written and oral information on study aims, research questions, context, confidentiality and dissemination principles.

### 3.5 Data Collection

Data were collected from a total of 17 experts in 12 individual interviews and two dyadic interviews from 2019 to 2020. Information power was deemed sufficient in relation to the study's aim, design and the quality of the interviews [57]. The interviews were performed by authors C.T., A.L., U.B., and a former researcher, and lasted between 23 min and 1 h 18 min with a mean of 48 min. The interviews began face‐to‐face at a safe location for the interviewee, but due to the COVID‐19 pandemic, they were later conducted online via Zoom meetings at Lund University (LU), using their IP address and secure password passages. The interviews were digitally recorded and transcribed verbatim by a research assistant with no prior contact with the participants. During the dyadic interviews, a moderator posed the main questions and invited further discussion before moving on. A co‐moderator observed the interaction and took field notes. An interview guide, developed by authors A.L. and U.B. in collaboration with a former researcher and service user representatives, was used throughout the data collection. The guide was applied across all stakeholder groups: service user experts, professionals, and managers. Sample questions included: ‘What, in your experience, are the top three priorities of mental health services?’, followed by ‘Why do you think these are?’ and ‘Would you prefer to prioritise them differently?’ A vignette, adapted to reflect the service user perspective (see Table 1), was presented to participants, followed by the question: ‘What does the gap look like to you between existing general MHSs and the proposed FACT idea?’ Using a vignette as a methodological tool enabled deeper exploration of how participants interpreted and attached meaning to the concept, allowing them to impose their own understanding during data collection [58]. The vignette provided sufficient context while leaving room for individual interpretation.

### 3.6 Data Analysis

**Figure 1:**
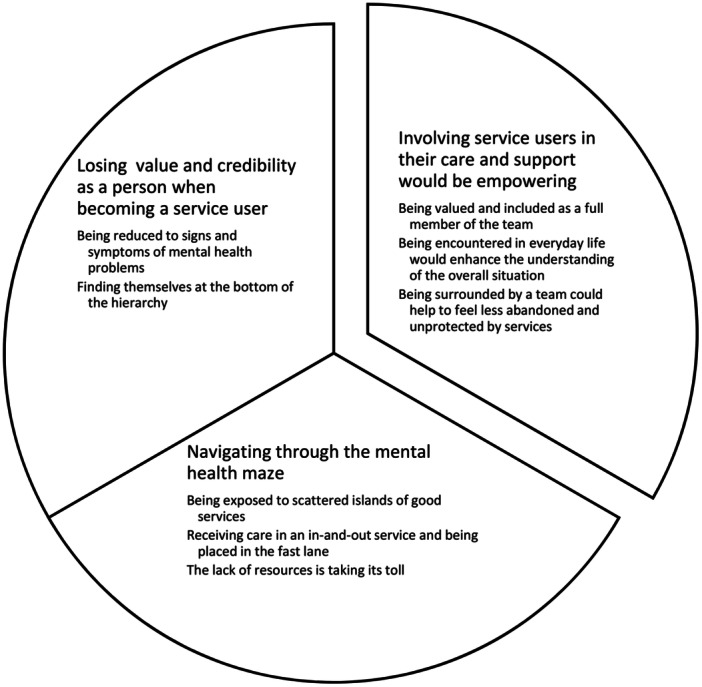
The results presented as three themes and related subthemes based on service users’ experiential knowledge of current specialist general mental health services. It also captures their reflections on the Flexible Assertive Community Treatment and its role in future practice.

Data were analysed using reflexive thematic analysis [49, 50]. The analysis allowed for in‐depth interpretation to capture the richness of the phenomenon, i.e., the experts' evaluation of current MHS and their reflections on the presented vignette presenting FACT, Table 1. First, each interview was familiarised by reading the transcripts several times and listening to the audio, a manifest coding was then performed separately by C.T., A.L. and E.N., and J.W. and U.B. Similar codes formed the subthemes and then themes were identified that corresponded to the research aim. This additional step was taken to ensure that the transcribed interviews were sufficiently accurate. Finally, the subthemes were combined into themes, which resulted in the overarching theme that corresponded to the more reflexive phase of the analysis (Table 2). For illustrative purposes, the experts in this study are renamed participants describing their current care, and their reflections on future MHS as service users in Section Results, 4. The authors E.N., C.T., J.W., and A.L. used an inductive, or bottom up, approach by identifying shared patterns of meaning within the interview transcripts. Prolonged engagement with the data was undertaken by the authors C.T., J.W., A.L. and U.B. Multiple interactions discussing the data analysis were performed by E.N., J.W., C.T., A.L. and U.B. with the aim of gaining theoretical coherence during the manifest—latent coding process see Supporting Information [59]. E.N. then developed a draft of the results (see Figure 1), which finalised the following interactions with J.W., C.T. and A.L. These processes were completed multiple times throughout the analysis and the writing of the manuscript, resulting in an iterative and reflexive process for the data analysis, and ensuring the credibility and trustworthiness of the process. All the authors were responsible for identifying the final themes and subthemes. Furthermore, to ensure credibility, EN performed text verification following the transcription by replaying the conversation audio files and validating them against the transcribed scripts.

**Table 2:** The initial codes and revisions were made during the data analysis process.

Extract 1	Initial coding	Reasons for revised coding	Revised coding
‘*Yes, but they can see if I'm getting worse, right? That way, I wouldn't have to contact the emergency department myself. I still fluctuate a lot; I'm both manic and suffering from depression. These conditions alternate persistently. In March, I had a long hypomanic period that lasted a month. It would have been nice if the housing support workers had been able to see it and call in, instead of me having to go to X and so on. That would have been great’*	Code: Mental health is fluctuating	There is an overlap between the frustration of knowing one's illness and the challenge that not all team members are familiar with the care plan 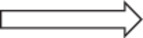	Code: Feeling frustrated knowing that they will need care, and not having strategies to handle it
	Supporting content: I still fluctuate a lot, and I'm both manic and suffering from depression		
	Code: Quite persistent		
	Supporting content: These conditions are quite persistent in each other		
	Code: Support workers to see it and call in	Substantial number of posts described that those working closest to the participants should have the knowledge required to support them 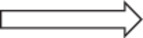	Code: Through a team‐work approach, all members would have the knowledge to support the service user
	Supporting content: It would have been nice if the support workers had been able to see it and call in		
	Code: Instead of me having to go to X and so on…		
	Supporting content: instead of me having to go to X and so on. That would have been great		
Extract 2	Initial coding	Reasons for revised coding	Revised coding
‘*But as I said, I've heard that others haven't had such positive experiences, so it may be that I've been a bit lucky too. I am actually satisfied with the care I've received. The good thing is that I have the same contact every time, so it feels like you get a certain continuity in care. For example, if I'd seen different psychiatrists each time, they would have to build a new relationship and get involved in one's care, which is difficult to do just based on a medical record compared to if you'd met them before. So, I think that's one of the big advantages of the care I've received’*	Code: Being a little lucky	Posts described a lack of belief in the possibility of receiving good care 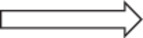	Code: Being exposed to scattered islands of good services can be challenging due to their inconsistent availability
	Supporting content: I have heard that others have not had positive experiences, so it may be that I have been a little lucky		
	Code: Big advantage with continuity of care	Several posts described that continuous care was essential, especially within a caring relationship 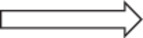	Code: Continuous care was essential for the participants to ensure their well‐being and support their ongoing needs
	Supporting content: Meeting the same psychiatrist each time is one of the big advantages of the care I have received		

#### 3.6.1 Service User Involvement

The study plan, ethical approval application, and interview questions were co‐produced with representatives from user organisations, professionals, and managers of MHS. One author contributed as both a service user and registered nurse, participating in the planning, data analysis, drafting and revising of the manuscript.

#### 3.6.2 Credibility and Reflexivity

Several strategies were carried out to enhance trustworthiness and quality of the study by using COnsolidated criteria for REporting Qualitative research checklist (COREQ) [60], as well as the Reflexive Thematic Analysis Reporting Guidelines (RTARG) [59]. Reflexivity was continuously applied, considering researcher–participant relationships and their potential influence on findings [61], alongside ongoing ethical dialogue within the team. The researchers brought diverse professional backgrounds, including public health nursing, occupational therapy, psychiatry, and clinical nursing. The first author (E.N.) is a PhD student with a licentiate degree in health sciences and a registered public health nurse. Collectively, the team's expertise spans mental health, public health, clinical medicine, recovery‐oriented interventions, integrated care, user involvement, co‐production and implementation research, using both qualitative methods and large‐scale trials.

## 4 Results

Service users' experiences of current care and reflections on the FACT vignette form three themes.

The first, *‘Losing value and credibility as a person when becoming a service user’*, describes experiences of encountering current MHS. The second, *‘Navigating through the mental health maze’*, captures their interactions with existing care. The third, *‘Involving service users in their care and support would be empowering’*, reflects responses to the vignette and thoughts on future needs. These themes and their subthemes are detailed below, supported by participant quotes.

### 4.1 Losing Value and Credibility as a Person When Becoming a Service User

This theme described participants experiences of encountering current services and is outlined in the two subthemes outlined below.

#### 4.1.1 Being Reduced to Signs and Symptoms of Mental Health Problems

Participants emphasised being human while living with complex MHP enduring fluctuating symptoms that varied in severity and challenged their ability to manage daily life. Co‐morbidity, comprising somatic, psychiatric, and substance‐related issues, added stress and further limited their ability to express themselves during encounters. Professionals treated mental health as separate from their physical health. Mental health professionals often struggled to assess additional symptoms. Participants felt reduced to a number, merely ticking a box. Services often ended abruptly, leaving them to manage care alone. A clear divide existed between those receiving care and those excluded. Services were incoherent; diagnoses varied depending on the professional consulted, creating additional complexity as each diagnosis required separate coordination.

Some professionals offered little space for dialogue and few opportunities for participants to share concerns about health or future care. At times, professionals openly debated psychiatric assessments in participants' presence, using language they barely understood. This fostered disbelief in professionals' motives, judgement and treatment. Participants perceived themselves as burdens and believed professionals saw them as encumbrances. Such experiences left participants feeling disempowered and disengaged from treatment. A clear mismatch emerged between standard service delivery and their needs. They believed recovery would be more likely if they were treated as persons rather than categorised by diagnoses or symptoms, current practices made them feel inhuman.

What I've been able to feel at times is that I'm… that I've been perceived as difficult, that there is something like; Oh my god, here she comes, self‐harm and there will be so much paperwork

Participant 10

#### 4.1.2 Finding Themselves at the Bottom of the Hierarchy

Participants expressed being a service user as occupying the lowest position in the hierarchy. Experiences of being treated as inhuman and disempowered reinforced feelings of exposure to a hierarchy within MHS. This hierarchy appeared in various forms but was consistently present. Unlike their experiences as of somatic care, it was communicated through subtle signs conveying unworthiness, leaving participants feeling discredited. They believed they were treated differently because of their mental health diagnoses, reflecting stigma.

Yes, that they, that physicians shouldn't, those in psychiatry and somatic care too, shouldn't be like a know‐it‐all, like they're masters of well‐being. That no, but “You can't have this and that”, “No but I think I know best here”, and “Don't think you're someone who knows better”.

Participant 5

This experience of being treated differently was also noted in differences in how the care environment was constructed for them as service users ‘*Then a nurse comes, with a recliner, a plastic chair, horrible’*. Overall, these encounters were experienced as impersonal, which created obstacles rather than support, sometimes necessitating additional treatment. Feelings of unworthiness translated into experiences of being unsafe, ignored, a nuisance, and explained to as if lacking understanding. Collectively, these experiences fostered a sense of abandonment and lack of protection.

### 4.2 Navigating Through the Mental Health Maze

This theme described participants process of finding tailored care and support for their MHPs, with three subthemes, described below.

#### 4.2.1 Being Exposed to Scattered Islands of Good Services

Participants emphasised that good MHS require professionals capable of building caring relationships. Such relationships enabled professionals to recognise needs, interpret the unspoken, and respond with respect and acceptance. Participants were disheartened that general MHS were not better equipped to offer this universally, despite its centrality to care.

Being met with respect and acceptance as human beings was fundamental. Their lived experience of MHP was valued alongside the health professionals' clinical expertise. Participants stressed the importance of contact with professionals who knew them well and could make informed decisions aligned with their wishes, particularly when self‐advocacy was difficult. Feeling listened to, included, and involved fostered a sense of support when most needed. They described receiving tailored treatment and timely care, before their condition hindered engagement in treatment or daily life. However, service quality varied. Not all professionals could build caring relationships, and exposure to isolated ‘islands of good care’ left participants feeling unsafe and uncertain about investing in support. Losing a trusted professional was described as overwhelming.

But I've noticed that as a service user you're rather… well, you're quite attached to the fact that if you encounter a health professional who is good, it's important that, that this person doesn't quit or become ill, and doesn't get pregnant. In other words, your commitment is tied to that health professional remaining as long as you are a service user yourself.

Participant 4

#### 4.2.2 Receiving Care in an In‐and‐Out Service and Being Placed in the Fast Lane

Participants described current care as being ‘placed in a fast lane’, resembling an in‐and‐out service focused on extinguishing crises and avoiding errors. Health professionals relied on standardised protocols, that is, suicide risk assessment, and the primary aim was symptom reduction through medication. Limited resources curtailed face‐to‐face time, and delayed follow‐ups negatively affected health. Professionals were experienced as only having time for essential tasks, leading to a loss of trust and belief in recovery.

Long waits for thorough assessment or treatment shifted the burden of care onto service users. Services were siloed, with poor collaboration between professionals and a knowledge gap between healthcare and municipal support. Participants expressed concern for frontline staff who saw them at their worst but lacked the means to help. Interprofessional dialogue was rare, and treatment goals often conflicted, even within the same organisation. This left participant feeling bounced between services and without control. Repeatedly recounting their story was exhausting.

… then the problem is that… that there are so many people in the health services, different physicians, different psychologists, and you get to meet different ones all the time, not the same psychiatrists. So, you have to (…) start and tell the same story again, over and over and over again, because it's new people. And that doesn't create security or reliability but chaos, and yes, makes it even worse.

Participant 3

#### 4.2.3 The Lack of Resources Is Taking Its Toll

Participants believed that limited resources contributed to shortcomings in care and support, and they therefore defended the professionals' situation. They described professionals as overloaded and stressed out. According to the participants, MHS that lacked financial resources and sufficient capacity for professionals to encounter service users were more fragile in terms of staff turnover. This economic system was considered to reinforce their experience of a non‐caring service that was delivering an inhuman, impersonalised and cynical care. Regional tendering was experienced as limiting their freedom of choice and therefore losing its value. The freedom of choice reform enabled the participants at the same time to change their address to get ‘good services’, which were otherwise restricted geographically. This was reflected on as ethically problematic since not all the service users had the abilities and/or resources to carry out such strategic planning to receive adequate care and support. There was a concern that this contributed to an unequal delivery of care and support, and the participants emphasised the need for the services to be inclusive for all service users. The prevailing culture of extinguishing fires increased the participants' feelings of being abandoned and unprotected. This thus impacted in turn on the reliability of the MHS in general and the participants' trust in them.

No, it'll be difficult and then… when the staff also suffer from mental health problems because they are so overstressed and exhausted, there'll be a lot of sick leave, and there'll be consequences for the service users…

Participant 9

### 4.3 Involving Service Users in Their Care and Support Would Be Empowering

This theme described the participants call for a collaborative and empowering and tailored care and support model where they, as service users, were included and equally valued for their expertise, which is described in the theme and the subthemes outlined below.

#### 4.3.1 Being Valued and Included as a Full Member of the Team

Participants stressed the need to be included as full team members, contrasting sharply with their current experience of MHS. Inclusion would enable open dialogue and shared responsibility for co‐creating solutions, grounded in professionals' acknowledgement that service users are experts on their mental health and life situation, reflecting person‐centredness. This description of shared decision‐making was seen as key to making services comprehensible. Participants suggested setting an overall goal as a team, agreeing on priorities, and working towards milestones. Such collaboration would also allow professionals to know service users better and challenge existing attitudes and assumptions.

So, I think that those who are more stable would know when they are feeling better, the support never disappears. They never have to search for services again, avoiding the whole merry‐go‐round.

Participant 2

Knowing that services were available daily, even when help was not immediately needed, would provide comfort and safety. Participants reasoned that this approach would improve self‐management, reduce service use and free resources for others. They believed everyday life, including employment, would become more manageable, fostering empowerment, belonging and value as contributing members of society. This shift was viewed as sustainable and financially beneficial for healthcare providers and society in general.

#### 4.3.2 Being Encountered in Everyday Life Would Enhance the Understanding of the Overall Situation

Participants believed professionals needed a deeper understanding of their overall situation and everyday context to grasp their mental health. Being met at home, as suggested in the vignette, evoked thoughts of MHS aligning more closely with reality and daily life. This outreach and ‘flexible approach’ were seen as challenging the existing hierarchy, as professionals would become guests in service users' homes, strengthening a holistic human approach and shifting attitudes.

Home visits were generally viewed positively, enabling tailored services, though participants expressed mixed feelings about inviting professionals into their private space. Structuring services around user involvement and standards for caring relationships would require professionals to assume new responsibilities, challenging current hierarchies and care culture. Participants feared resistance from those reluctant to relinquish power, which could undermine sustainability. They also noted that interventions demand resources and time. This was experienced as currently lacking, making it difficult to persuade professionals to participate, especially if it disrupts established norms.

So, it's very far away from one's own reality when you go there… To be met in one's everyday life I think is very important, because it's everyday life that you must manage and function in, and that you almost need to bring support home as well, like.

Participant 1

#### 4.3.3 Being Surrounded by a Team Could Help to Feel Less Abandoned and Unprotected by Services

Participants found the team‐based FACT approach appealing, believing it would benefit both service users and professionals. It promised continuity of care, availability and sustainable knowledge development. Most importantly, they would not fear ‘letting go of care’, knowing a whole team was available if needed. Continuity was considered critical, and participants wished for professionals to walk alongside them in a caring relationship. They suggested developing structures that support continuity and caring relationships.

A team approach was valued for offering multiple perspectives, reducing the burden on individual professionals, and easing stress. It was expected to bridge gaps between services, foster collaboration on prevention and health promotion, and create opportunities for mutual learning. Teams could also improve timing and delivery of care, tailoring support to users' needs.

Yes, exactly. If you'd worked in a team, not just one person would have handled it, but someone with time could have taken over. That would have been a big advantage. The point of care is for service users to feel better and be healthier.

Participant 11

## 5 Discussion

This study explores service users' experiences of current specialist general MHS and their reflections on FACT as a recovery‐oriented care model. It highlights a call for future services that adopt an empowering approach, viewing individuals as capable. In contrast, current specialist MHS were often experienced as failing to meet the needs of those with complex MHP, underpinned by a default perception of incapability. A Swedish study on completed suicides found that over half of reported deficiencies related to lack of treatment and inadequate suicide assessment, while a third concerned poor external communication [62]. Effective communication is essential to delivering care grounded in the belief that service users are capable. Being supported through caring relationships and receiving flexible, continuous and structured services was seen as vital [16]. Caring relationships stems from caritas, an act of love and mercy [31]. Integration of caring (illness experience) and medicine (disease treatment) enables health promotion [8]. It is nor confined to any one profession, though associated with nursing [31].

Participants noted that traditional expert, that is, health professional‐patient roles remain deeply rooted, as described in *‘Losing value and credibility as a person when becoming a service user’*. Historically, the service user movement encouraged individuals to move beyond passive opinion‐giving and become active agents of change [63]. This shift was shaped by developments in service provision, deinstitutionalisation, the legitimacy of biomedical models, and the rise of consumerism. Service user involvement has since evolved to encompass five key attributes: person‐centred care, informed decision‐making, advocacy, gathering user feedback, and partnership working. However, participants described traditional roles as hindering collaboration, disrupting continuity, and limiting caring relationships, resulting in fragmented and inconsistent quality [64].

One major barrier to delivering person‐centred specialist MHS is the persistence of these roles [65, 66], where professionals retain power to decide what is best for patients [65]. Health professionals view this as part of their duty and disciplinary boundaries. Guarding traditional practices and stereotypical attitudes further obstructs person‐centred care, echoing participants' view that this is the greatest challenge to changing current MHS practices [67].

While stigma can be reduced using educational initiatives [68, 69], another form of reducing stigma is by exposing professionals to experiential knowledge of service users [70]. One such intervention is the implementation of recovery‐oriented, integrated care and support models [71]. Implementing a person‐centred approach within psychosis inpatient care improved the working environment, enabled more flexible care routines, and enhanced interactions with service users [68]. Embedding the ethics of person‐centred care offers a way to challenge traditional practices and roles within services. Being taken seriously and listened to with an understanding attitude is essential to person‐centred care [9, 10], while the absence of such recognition can have serious consequences for service users' health [23, 38, 62, 72, 73]. This understanding attitude requires professionals to view service users as full partners, capable of making active decisions about their care and support [8, 68, 69]. Moving from siloed care to a team‐based approach, as described in *‘Being valued and included as a full member of the team’* and *‘Being surrounded by a team would help to feel less abandoned and unprotected by services'*, was seen as a way to address the power imbalance between service users and professionals. According to our results, there is a need for greater collaboration to inclusively tailor the care and support in relation to service users' everyday life context to gain a better understanding of the overall situation. The service users' navigation through a mental health maze could thus be facilitated by involving services users in their care and support, as referred to in the themes.

The subtheme ‘*The lack of resources is taking its toll’* highlighted how organisational constraints within MHS hinder professionals from delivering care aligned with service users' needs. Sweden's primary healthcare freedom‐of‐choice reform has disadvantaged vulnerable patients with complex needs, such as those with MHPs [48]. Private providers often prioritise financial incentives, and MHS incur higher daily costs than somatic care [74]. Policymakers must develop financial structures, and providers must organise services to meet users' needs. Research shows that health professionals adapt to financial pressures, categorising patients' needs to fit booking systems and delivering care at inappropriate levels, echoing participants' accounts [75]. This suggests a conflict between providers' costs and available resources, limiting services and leaving some users unsupported when needed.

Service users' signs and symptoms of MHP may vary over time [21, 76] which was also described by participants in this study. Feelings of distress, not knowing what is happening to one's body, and a helplessness when in contact with services, including primary MHS [76, 77, 78, 79], and services related to co‐morbidity [39, 80]. The service users' right to health is viewed as profoundly important for perceived health care quality from a human rights perspective [81]. Improvements to current care were considered urgent and necessary by the participants. They called for greater collaboration to provide empowerment, tailored care and support, where they, as service users, were equally valued and acknowledged for their expertise of their perceived health by the health professionals. It is crucial for providers to reflect on power imbalances to enable effective partnerships in MHS [82]. Thus, exploring mental health providers experiences and reflections on current care is essential before implementing an integrative, recovery‐oriented care and support model such as FACT.

The participants have described deficiencies in financial resources as a key restriction in building collaborative, empowering, and inclusive tailored care and support. Therefore, there is a need for future research to include cost‐effectiveness when aiming to implement an integrative, recovery‐oriented care and support model. It may also be necessary for future research to include the ethics of person‐centred care, where the encounters between service users (perceived illness) and professionals stem from the inevitability of forming partnerships [10]. Service users' subjective perception of mental health needs is a better predictor for forecasting future MHS than objective assessments [83]. The use of experiential knowledge in research should thus be viewed as an important element for leading the direction of care [5, 84] and informing health professionals' evidence‐based practice [35].

## 6 Trustworthiness and Limitations

All researchers contributed to interpreting the phenomenon, its development, and significance for participants, while remaining close to the narrative [50]. Authors E.N., C.T., J.W., A.L. and U.B. led the analysis, engaging in repeated discussions to ensure theoretical coherence and inform revised coding [59]. Preunderstandings and mental health expertise were acknowledged in the *Service User Involvement and Reflexivity Statement* and *Study Design*. Reflections from diverse professional backgrounds further shaped interpretation. Participants, self‐identified experts, consented voluntarily, which may affect representativeness. Member checking was not feasible, limiting deeper insight. Conducted during the COVID‐19 pandemic, participants' extensive service experience meant they were not ‘new’ to the field; their accounts should not be attributed solely to the pandemic. Due to topic sensitivity, focus groups were replaced with individual and dyadic interviews, considered ethically appropriate and enabling rich, experience‐based data [51]. Personal characteristics were not collected, in line with design and confidentiality principles [59]. Participants' experiential knowledge was recognised as uniquely valuable [5].

## 7 Conclusions and Clinical Implications

In conclusion, the participants call for greater collaboration to empower and provide inclusive tailored care and support, which they emphasised as essential for the future of MHS. Further, the participants described the difficulty for service users to navigate in the current general MHS and by involving them as service users, care and support would be empowering. The clinical implications are that there is a need to integrate the service users' experiential knowledge into clinical practices, to create an evidence‐based practice [35].

## Author Contributions

**Emmy Nilsson:** formal analysis (lead), methodology (equal), project administration (lead), validation (lead), visualisation (lead), writing – original draft preparation (lead), writing – review and editing (equal). **Carina Tjörnstrand:** investigation (equal), writing – review and editing (equal), formal analysis (supporting), validation (supporting), writing – review and editing (supporting). **Daniel Lindqvist:** conceptualisations (equal), funding acquisition (equal), resources (equal), writing – review and editing (supporting). **Jenny Wetterling:** conceptualisation (supporting), formal analysis (supporting), validation (supporting), writing – review and editing (supporting). **Annika Lexén:** conceptualisation (supporting), investigation (equal), methodology (equal), formal analysis (supporting), supervision (supporting), validation (supporting), writing – review and editing (equal). **Ulrika Bejerholm:** conceptualisation (equal),funding acquisition (equal), investigation (equal), methodology (equal), formal analysis (supporting), resources (equal), supervision (lead), validation (supporting), writing – original draft (supporting), writing – review and editing (equal).

## Ethics Statement

Ethical approval was sought and provided by The Swedish Ethical Review Authority, Dnr 2019‐02866. The research was performed in accordance with the Swedish Ethical Review Act [85] and the ethical practice outlined in ‘Declaration of Helsinki: Ethical Principles for Medical Research Involving Human Participants' [86].

## Consent

The participants have given their informed consent to publish this study.

## Conflicts of Interest

The authors declare no conflicts of interest.

## Data Availability

The data that support the findings of this study are available on request from the corresponding author. The data are not publicly available due to privacy or ethical restrictions.
